# Oxylipins From Different Pathways Trigger Mitochondrial Stress Signaling Through Respiratory Complex III

**DOI:** 10.3389/fpls.2021.705373

**Published:** 2021-07-29

**Authors:** Yovanny Izquierdo, Luis Muñiz, Jorge Vicente, Satish Kulasekaran, Verónica Aguilera, Ana López Sánchez, Ada Martínez-Ayala, Bran López, Tomás Cascón, Carmen Castresana

**Affiliations:** ^1^Department of Plant Molecular Genetics, Centro Nacional de Biotecnología (CNB-CSIC), Madrid, Spain; ^2^School of Biosciences, University of Nottingham, Nottingham, United Kingdom

**Keywords:** arabidopsis, oxylipins, mitochondria, Antimycin A, reactive oxygen species, complex III, retrograde signaling

## Abstract

Plant oxylipins are signaling molecules produced from fatty acids by oxidative pathways, mainly initiated by 9- and 13-lipoxygenases (9-LOX and 13-LOX), alpha-dioxygenases or non-enzymatic oxidation. Oxylipins from the 9-LOX pathway induce oxidative stress and control root development and plant defense. These activities have been associated with mitochondrial processes, but precise cellular targets and pathways remain unknown. In order to study oxylipin signaling, we previously generated a collection of *Arabidopsis thaliana* mutants that were insensitive to the 9-LOX products 9(*S*)-hydroxy-10,12, 15-octadecatrienoic acid (9-HOT) and its ketone derivative 9-KOT (*noxy* mutants). Here, we describe *noxy1, noxy3, noxy5, noxy23*, and *noxy54* mutants, all affected in nucleus-encoded mitochondrial proteins, and use them to study the role of mitochondria in oxylipin signaling. Functional and phenotypic analyses showed that *noxy* plants displayed mitochondrial aggregation, reduced respiration rates and resistance to the complex III inhibitor Antimycin A (AA), thus indicating a close similarity of the oxylipin signaling and mitochondrial stress. Application of 9-HOT and 9-KOT protected plants against subsequent mitochondrial stress, whereas they boosted root growth reduction when applied in combination with complex III inhibitors but did not with inhibitors of other respiratory complexes. A similar effect was caused by linear-chain oxylipins from 13-LOX or non-enzymatic pathways having α,β-unsaturated hydroxyl or keto groups in their structure. Studies to investigate 9-HOT and 9-KOT activity indicated that they do not reduce respiration rates, but their action is primarily associated with enhanced ROS responses. This was supported by the results showing that 9-HOT or 9-KOT combined with AA amplified the expression of oxylipin- and ROS-responding genes but not of the AA marker *AOX1a*, thus implying the activation of a specific mitochondria retrograde signaling pathway. Our results implicate mitochondrial complex III as a hub in the signaling activity of multiple oxylipin pathways and point at downstream ROS responses as components of oxylipin function.

## Introduction

The term oxylipin is generically used to group oxidized lipid derivatives produced from the oxidation of fatty acids. Oxylipins are ubiquitous throughout life kingdoms and usually have signaling functions. In animals, prostaglandins and leukotrienes mediate inflammatory responses (Haeggström and Funk, 2011), whereas in bacteria, oleic-acid derived oxylipins participate in quorum sensing and virulence (Martínez and Campos-Gómez, 2016). In plants, hundreds of different oxylipins have been reported, but there are vast differences in our knowledge of different existing pathways (Wasternack and Feussner, 2018).

**Figure 1:**
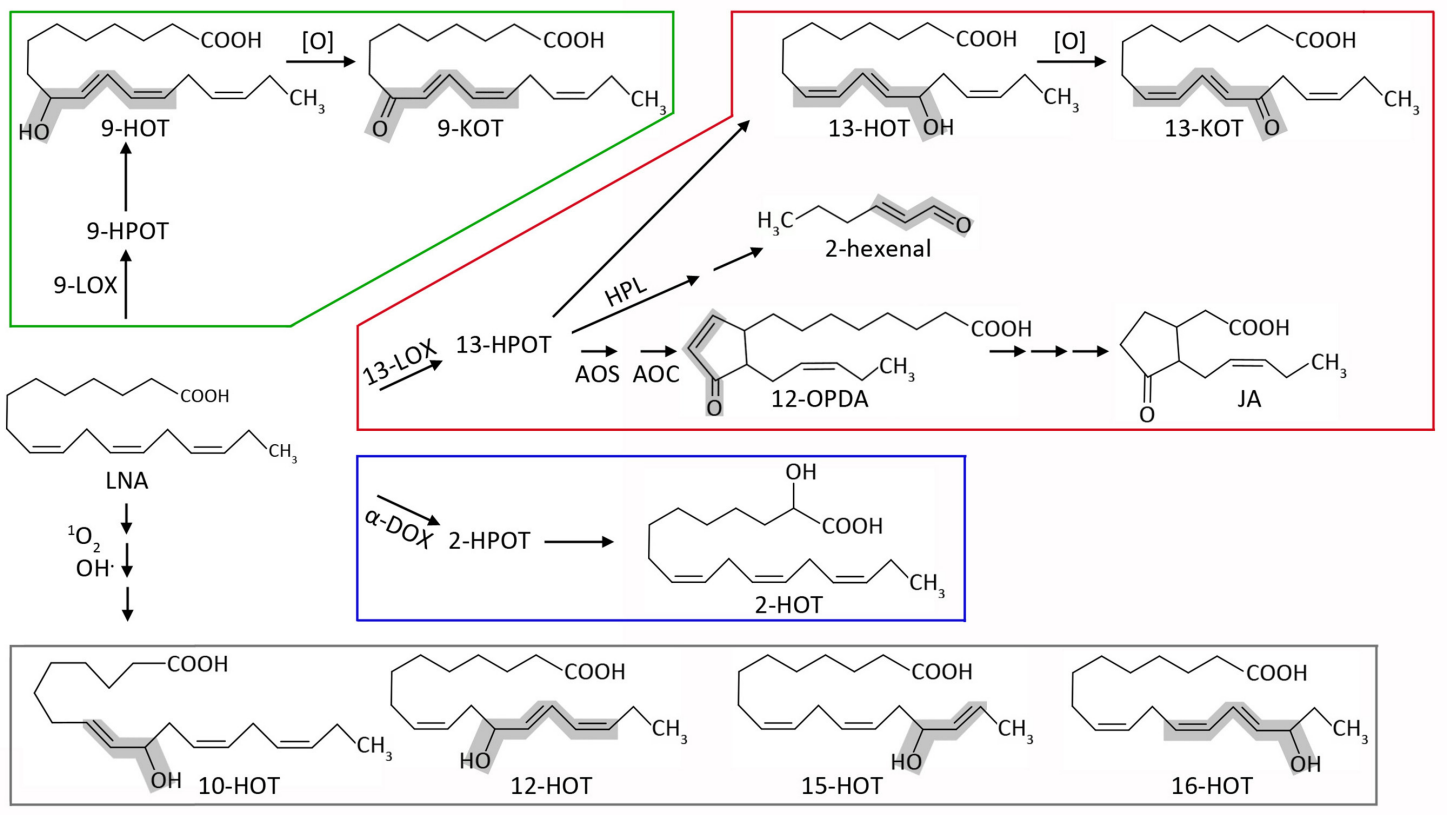
Schematic representation of the major oxylipin biosynthesis pathways, focused on the derivatives used in this work. Starting from linolenic acid (LNA), oxygenation can be carried out by 9-lipoxygenases (9-LOX, green), 13-lipoxygenases (13-LOX, red), α-dioxygenases (α-DOX, blue), or non-enzymatic reactions (gray). Lipoxygenases generate the hydroperoxides 9-HPOT and 13-HPOT, which are then reduced to hydroxyl-acids 9-HOT and 13-HOT. These can be further oxidized to 9-KOT and 13-KOT (alternatively, lipoxygenases could directly generate keto-acids from hydroperoxides, which is not shown for simplicity). 13-HPOT can be fragmented by hydroperoxide lyase (HPL) to 2-hexenal or committed to the jasmonate pathway by the sequential action of allene oxide synthase (AOS) and allene oxide cyclase (AOC) leading to 12-OPDA. The latter can be reduced and subjected to three β-oxidation cycles leading to jasmonic acid (JA). α-dioxygenases mainly yield 2-HOT *in vivo*, also through a hydroperoxide intermediate (2-HPOT). LNA can also react directly with singlet oxygen or hydroxyl radical. This pathway can lead to 9-HOT and 13-HOT (not shown), but also to oxylipins exclusively generated by non-enzymatic reactions (10-HOT, 12-HOT, 15-HOT and 16-HOT). α,β-unsaturated carbonyl (RES) or hydroxyl (RES precursor) moieties are highlighted in gray. For simplicity, stereochemistry details have been omitted (e.g., 13-LOX and 9-LOX yield *S*-enantiomers whereas non-enzymatic reactions produce racemic mixtures) as well as additional oxylipins generated by secondary enzymatic or non-enzymatic reactions.

Plant oxylipins are produced mainly during stress responses. Biosynthesis occurs by both enzymatic or non-enzymatic incorporation of oxygen, mainly to polyunsaturated fatty acids such as linoleic (18:2, LA) or linolenic acid (18:3, LNA). Figure 1 shows a schematic representation of the major oxylipin biosynthesis pathways, focused on the derivatives used in this study. The first step of oxylipin synthesis is usually the formation of a lipid hydroperoxide, followed by secondary modifications leading to a vast array of compounds (Wasternack and Feussner, 2018). Depending on the position of the initial oxygenation, several enzymatic pathways can be defined. Taking LNA as initial substrate, α-dioxygenases (α-DOX) lead to 2(*R*)-hydroxy-9(*Z*),12(*Z*),15(*Z*)-octadecatrienoic acid (2-HOT, Hamberg et al., 2003), whereas 9- and 13- lipoxygenases (9-LOX and 13-LOX) catalyze stereospecific oxidation in positions 9 and 13, respectively. Hydroperoxides derived from LNA and 13-LOX can be either committed to jasmonic acid synthesis or transformed into 13(*S*)-hydroxy-9(*Z*),11(*E*),15(*Z*)-octadecatrienoic acid (13-HOT) and 13-keto-9(*Z*),11(*E*),15(*Z*)-octadecatrienoic acid (13-KOT). Likewise, 9-LOX activity generates mainly 9(*S*)-hydroxy-10(*E*),12(*Z*),15(*Z*)-octadecatrienoic acid (9-HOT) and 9-keto-10(*E*),12(*Z*),15(*Z*)-octadecatrienoic acid (9-KOT; Blée, 2002; Andreou and Feussner, 2009; Mosblech et al., 2009). LNA peroxidation can also take place non-enzymatically during severe oxidative stress, by reaction with singlet oxygen or hydroxyl radicals. In these cases, oxygen addition can occur at any unsaturated carbon, leading to HOT and possibly KOT derivatives in positions 9, 10, 12, 13, 15, and 16 (Göbel et al., 2003; Mosblech et al., 2009). Lipid fragmentation is also common, leading to short chain α,β-unsaturated aldehydes or ketones such as malondialdehyde, 2-hexenal and acrolein (Alméras et al., 2003; Farmer and Mueller, 2013; Mochizuki et al., 2016). These reactive electrophilic species (RES) are considered universal lipid peroxidation markers and can bind covalently to cysteine residues (Mueller and Berger, 2009; Ameye et al., 2018). Some lipoxygenase products, such as 9-KOT and 13-KOT, also contain α,β-unsaturated carbonyl, but little is known about their specific reactivity.

13-LOX-derived jasmonic acid is by far the most studied oxylipin. As a key hormone controlling both developmental and stress-related processes, its receptor, co-receptors, and downstream-regulated transcription factors and target genes are well defined (Chini et al., 2009; Ruan et al., 2019). In addition to jasmonates, oxylipins from the 9-LOX and non-enzymatic pathways are also implicated in plant stress responses. In Arabidopsis roots, 9-HOT induces a characteristic waving phenotype, accompanied by oxidative stress and callose deposition (Vellosillo et al., 2007). These processes are mediated by brassinosteroid signaling and contribute to defense against pathogens (Marcos et al., 2015). In leaves, 9-HOT and 9-KOT (and other hydroxy and keto derivatives of LNA) accumulate in response to infection with avirulent bacteria, contributing to the hypersensitive response (HR) in different plant species (Rustérucci et al., 1999; Göbel et al., 2002; Jalloul et al., 2002; Battilani et al., 2018). Moreover, inoculation of 9-KOT induces local and systemic acquired resistance (SAR) against subsequent *Pseudomonas* infection (Vicente et al., 2012). Oxylipins produced exclusively by non-enzymatic reactions (e.g., 10, 12, 15, and 16-HOT) have also been detected in strong oxidative conditions leading to lipid peroxidation, like the onset of HR and cell death (Göbel et al., 2003), but very little is known about their functions.

Despite abundant evidence on 9-LOX-oxylipin functions in plant responses, their precise perception and signaling pathways remain elusive. In an attempt to identify specific signaling pathways, Walper et al. (2016) identified transcription factors involved in 9-HOT response, but concluded these were mostly related to oxylipin detoxification instead of signal transduction. In a previous study, we took advantage of the waving phenotype induced by 9-HOT to generate a collection of non-sensitive to oxylipin (*noxy*) mutants (Vellosillo et al., 2007). Three of these signaling mutants (*noxy2, noxy15/drp3a-1* and *noxy38/fmt-1*) displayed abnormal mitochondrial aggregates and were affected in mitochondrial stress responses (Vellosillo et al., 2013). In the same study, 9-HOT was found to decrease the mitochondrial membrane potential, collectively suggesting that 9-LOX oxylipin signaling was associated to mitochondrial processes.

Here, we describe five additional *noxy* mutants (*noxy1, noxy3, noxy5, noxy23*, and *noxy54*), all affected in mitochondrial distribution and function, and at the same time resistant to Antimycin A (AA), a mitochondrial complex III (C-III) inhibitor commonly used to study mitochondrial retrograde signaling (Yu et al., 2001). Studies with mitochondrial respiration inhibitors in combination with 9-HOT and 9-KOT, showed that these oxylipins exacerbate the root growth arrest caused by inhibitors of C-III, but not of other respiratory complexes. This effect was associated to an increment of ROS production and changes in stress gene expression. The application of 9-HOT and 9-KOT protected plants against subsequent mitochondrial stress. These activities were extensive not only to 9-LOX derivatives, but also to structurally related oxylipins from 13-LOX and non-enzymatic pathways (collectively named here as mitochondria-active oxylipins) suggesting a common mitochondrial effect. Altogether, our results point to mitochondrial C-III as a hub in oxylipin signaling that would cause mild mitochondrial damage to trigger a stress response protecting plants against cellular injury.

## Materials and Methods

### Plant Material, Mutant Characterization and Growth Conditions

*Arabidopsis thaliana* wild type and mutant plants were derived from Columbia-0 ecotype. *noxy1, noxy3, noxy5, noxy23*, and *noxy54* mutants were generated in a previous screening of an EMS-mutagenized population (Vellosillo et al., 2007). Therein, *noxy* mutants were selected due to their inability to show a root waving phenotype in presence of 9-HOT. To identify mutations, *noxy* plants were crossed to wild type Landsberg erecta ecotype and F2 mutants were selected. DNA from *noxy* and 400 recombinant mutants was used to analyze mutation linkage as described by Ponce et al. (2006). Massive genome sequencing performed at BGI genomics (http://www.genomics.cn) was used to identify *noxy* mutations in the mapped region. For microscopy analysis, Col-0;*35S:mtYFP* plants (Nelson et al., 2007; Vellosillo et al., 2013) were crossed with *noxy* mutants. Additional mutants used were *ahg2-1*(Nishimura et al., 2004) and T-DNA insertion lines *lon1-4* (SALK_013817) and *noxy23-2* (SALK_036918) which were obtained from the Nottingham Arabidopsis Stock Center (Nottingham, UK).

For *in vitro* analysis, seeds were sterilized with 75% bleach and vernalized for 3 days. Germinated seedlings were grown vertically in controlled chambers at 22°C with 14 h light (120 μmol s^−1^m^−2^) and 10 h dark, in fluorescent illumination. Culture medium was 0.5 × MS with 1.5% (w/v) agar and 1.5% (w/v) sucrose. For phenotype analysis, seedlings were transferred after 4 days to the same medium supplemented with specific products at the indicated concentrations. In pretreatment assays, plants were germinated directly in oxylipins (25 μM), LNA (25 μM) or AA (2.5 μM) and transferred after 4 days to AA-containing (15 μM) MS medium. In all cases, root growth was measured 3 days after transference to the new medium. For gene expression analysis, 7-day-old seedlings were moved to liquid medium (0.5 × MS with 1.5% sucrose), kept overnight for acclimation, and treated with oxylipins (20 μM), AA (20 μM), or their combinations.

### Reagents and Chemicals

Oxylipins used in this study (Supplementary Table 1) were obtained as previously described. 9-LOX and 13-LOX derivatives (9-HOT, 9-KOT, 13-HOT, and 13-KOT) were produced as in Prost et al. (2005); α-DOX derivative 2-HOT was synthesized as in Hamberg et al. (1999); non-enzymatically produced 10-HOT, 12-HOT, and the mixture 15-HOT/16-HOT, were obtained by singlet oxygen oxygenation (Przybyla et al., 2008). Oxylipin stocks were prepared in 95% ethanol and diluted in buffer or culture media to reach appropriate concentrations. OPDA and JA were purchased from Larodan Fine Chemicals. Additional products used for phenotype analysis and respiration assays were purchased from Sigma Aldrich with at least 95% purity: Antimycin A (A8674), Myxothiazol (T5580), Rotenone (R8875), Carboxin (45371), Sodium malonate (63409), KCN (60178), Oligomycin A (75351), Sodium ascorbate (A7631), *tert*-butyl hydroperoxyde (458139), 2(*E*)-hexenal (132659), Paraquat (36541), NADH (10107735001), ATP (A1852), ADP (01905), Sodium succinate (S2378), Dithiothreitol (D0632) and n-Propyl gallate (P3130).

### Root Phenotype Analysis

Vertically grown plants were photographed and root lengths were measured with Fiji software (https://fiji.sc/). For each genotype, measurements were presented as relative root growth with respect to plants grown in control conditions. In single treatments, statistical differences were determined by Student's *t*-test. Interaction in combined treatments was determined by two-way ANOVA with R functions “aov” and “Anova.”

### Confocal Microscopy

Mitochondrial distribution in wild type and *noxy* plants expressing mt-YFP was observed in the elongation zone of 7-day-old seedling roots. Images were taken with a Leica Stellaris microscope using a 20 × objective and 4 × zoom amplification. A 488 nm laser line was used for excitation, and fluorescence was detected in the range 500-636 nm. Localization of NOXY1:GFP was determined by co-localization with the mitochondrial marker mt-mCherry in *Nicothiana benthamiana* leaves. NOXY1:GFP construct was prepared by cloning the genomic sequence of NOXY1 into the pGWB5 vector, and it was transiently coexpressed with mt-mCherry (Nelson et al., 2007) in *N. benthamiana* by Agrobacterium-mediated transformation. Images were taken with a Leica TCS-SP8 microscope using a 63x objective. Sequential scanning was done with 488 nm (for GFP) and 575 nm (for mCherry) excitation laser lines. Fluorescence emission bands 496–550 and 585–660 nm were used to detect GFP and mCherry, respectively. Raw images were exported to jpeg format with LASX software (https://www.leica-microsystems.com/).

### Mitochondrial Isolation

Mitochondria were isolated as described by Whelan and Murcha (2015). Briefly, 10 g of 2-week-old, plate-grown plants were collected and immediately ground with 7 g of sea sand in 40 ml of cold extraction buffer (0.3 M sucrose, 25 mM tetrasodium pyrophosphate, 10 mM KH_2_PO_4_, 2 mM EDTA, 1% w/v PVP-40, 1% w/v defatted BSA, 18 mM sodium ascorbate, 20 mM cysteine, pH 7.5). Homogenate was filtered and centrifuged at 2,450 × *g*. Supernatant was collected and centrifuged 20 min at 17,400 × *g*. The resultant pellet was collected by gently resuspending in 1 ml wash buffer (0.3 M sucrose, 10 mM TES, 0.1% defatted BSA, pH 7.5) using a paintbrush. This suspension contained crude mitochondria and chloroplasts, and was cleaned up by repeating both previous centrifugation steps, and finally resuspended in 400 μl of wash buffer. The resulting chloroplast/mitochondria preparation was overlaid in continuous PVP-40/Percoll gradients and ultracentrifuged in a SW40Ti rotor (Beckman Coulter) for 40 min, at 40,000 × *g* (no brake). Mitochondria formed a yellowish band close to the bottom. They were carefully aspirated, placed in a new ultracentrifuge tube, and washed by filling with wash buffer and centrifuging 20 min at 31,000 × *g* with light brake. This washing step was repeated with 15 min centrifugation at 18,000 × *g* and supernatant was carefully removed. The mitochondrial pellet was collected by aspiration with a Pasteur pipette in a minimum volume (~200 μl). Protein concentration was measured with Bio-Rad Protein Assay kit (Bio-Rad).

### Respiration Measurement

Mitochondria respiratory capacity was estimated by measuring oxygen consumption of purified mitochondria (30–50 μg protein) with a Clark electrode (Oxygraph, Hansatech) using 5 mM sodium succinate and 1 mM NADPH as substrates, as described by Whelan and Murcha (2015).

### Western Blot

Western blot was performed by standard procedures. Samples were extracted in 4 M urea, 50 mM TrisHCl pH 7.5, 150 mM NaCl, 0.1% (w/v) Nonidet P40, 1 mM PMSF and 1X complete protease inhibitor cocktail (Roche). Protein-containing supernatants were collected by centrifugation (10,000 × *g*, 10 min, 4°C). 25 μg protein per sample were separated by SDS-PAGE in 10% acrylamide gels, transferred to nitrocellulose membranes (GE Health Care), stained with red Ponceau (0.1%), washed, blocked with 5% defatted powder milk in TBS and incubated with an antibody specific to plant AOX1/2 (Agrisera) diluted 1:1000 in TBS with 1% defatted powder milk. Blots were then incubated with a peroxidase-labeled anti-rabbit antibody (Agrisera) and revealed with ECL Select^TM^ (Amersham). Blot images were taken in a ChemiDoc^TM^ imaging system (BioRad).

### RNA Isolation and RT-qPCR

For each treatment, RNA was isolated from three biological replicates by Guanidine hydrochloride/Phenol-chloroform extraction (Logemann et al., 1987). Contaminant DNA was removed using Turbo DNA-free^TM^ kit (ThermoFisher). cDNA was synthesized with Transcriptor First Strand cDNA Synthesis Kit (Roche) using hexamer random primers and RNA denaturation at 60°C. qPCR was performed in a 7500 thermocycler (Applied Biosystems), using NZYSpeedy qPCR Green Master Mix (2x) and specific primers (Supplementary Table 2). Expression was normalized combining 18S rRNA and *SAND* gene (At2g28390, described in Czechowski et al., 2005) as references.

## Results

### Identification of *noxy* Mutants With Mitochondrial Dysfunction

Previous studies with the 9-LOX product 9(*S*)-hydroxy-10,12,15-octadecatrienoic acid (9-HOT) demonstrated the importance of mitochondria in 9-LOX-derived oxylipin signaling (Vellosillo et al., 2013; Marcos et al., 2015). 9-HOT caused mitochondrial aggregation and loss of inner membrane potential when added to wild type plants, whereas 9-HOT insensitive mutants *noxy2, noxy15/drp3a-1* and *noxy38/fmt-1* exhibited constitutive mitochondrial alterations (Vellosillo et al., 2013).

The activity of 9-HOT on mitochondria was further investigated here by using additional *noxy* mutants (*noxy1, noxy3, noxy5, noxy23*, and *noxy54*). They all had shorter roots than wild type plants and were impeded in characteristic 9-HOT responses such as root waving and callose deposition (Supplementary Figure 1), similar to other oxylipin-insensitive *noxy* mutants (Vellosillo et al., 2007, 2013; López et al., 2011; Marcos et al., 2015; Izquierdo et al., 2018).

A combination of classical map-based positional cloning and next-generation sequencing allowed the identification of *noxy* mutations, summarized in Table 1. This genetic analysis indicated that all *noxy* mutations were monogenic and recessive. *noxy1* and *noxy5* were located at loci At1g43980 and At3g02010, respectively. Both encode mitochondrial Pentatricopeptide Repeat (PPR) proteins, which are usually associated with post-transcriptional processing of chloroplast or mitochondrial RNA (Lurin et al., 2004). The *noxy1* mutation is a G-to-A transition between repeats 11 and 12 that changes Gly-366 to Glu in the protein sequence. The *noxy5* mutation is a C-to-T transition that converts Thr-227, within repeat 6, to Met. *noxy3* was located at locus At5g26860 encoding the mitochondrial protease LON1, involved in the control of protein turnover (Rigas et al., 2009). *noxy3* mutant contains two G-to-A nucleotide mutations in *LON1* gene, changing Arg-441 and Glu-444 to Lys residues. As mutant alleles *lon1-1* and *lon1-2* were previously described (Solheim et al., 2012), *noxy3* was renamed as *lon1-3*. The *noxy23* mutation is a G-to-A transition changing Glu-199 to Lys in locus At1g64880, which encodes a member of the S5 ribosomal protein family with mitochondrial localization (Bonen and Calixte, 2006). Finally, mutation *noxy54* was located at the locus At1g55870 encoding AHG2 (ABA Hypersensitive Germination 2), a mitochondrial poly(A)-specific exoribonuclease (AtPARN) that controls mRNA levels in this organelle (Hirayama et al., 2013). *noxy54* plants contain a G-to-A transition that turns Trp-552 into a stop codon and produces a truncated protein. As mutant alleles *ahg2-1* and *ahg2-2* were previously characterized (Nishimura et al., 2004; Hirayama et al., 2013), *noxy54* was renamed as *ahg2-3*.

**Table 1:** Mutants used in this study.

**Mutant**	**Renamed as**	**Gene name**	**AGI**	**Localization**	**Description (Uniprot)**
*noxy1*	*noxy1*	*NOXY1*	At1g43980	Mitochondria^a^^,^^e^	PPR-containing protein, mitochondrial
*noxy3*	*lon1-3*	*LON1*	At5g26860	Mitochondria^b^	Lon protease homolog 1, mitochondrial
*noxy5*	*noxy5*	*NOXY5*	At3g02010	Mitochondria^a^	Putative PPR-containing protein
*noxy23*	*noxy23*	*NOXY23*	At1g64880	Mitochondria^c^	Ribosomal protein S5 family protein
*noxy54*	*ahg2-3*	*AtPARN*	At1g55870	Mitochondria^d^	Poly(A)-specific ribonuclease (PARN)

a *Lurin et al. (2004)*,

b *Ostersetzer et al. (2007)*,

c *Bonen and Calixte (2006)*,

d *Hirayama et al. (2013)*,

e *This study*.

NOXY3/LON1, NOXY5, NOXY23, and NOXY54/AHG2 had been demonstrated to be nuclear-encoded mitochondrial proteins (Lurin et al., 2004; Bonen and Calixte, 2006; Ostersetzer et al., 2007; Hirayama et al., 2013), whereas experimental evidence for subcellular localization of *NOXY1* gene product was still lacking. To examine NOXY1 cellular localization, we transiently coexpressed a *35S:NOXY1-GFP* construct together with the mitochondrial marker mt-mCherry in *N. benthamiana* leaves. Fluorescence microscopy showed that GFP and mt-mCherry colocalized, supporting the mitochondrial localization of NOXY1 protein (Supplementary Figure 2).

**Figure 2:**
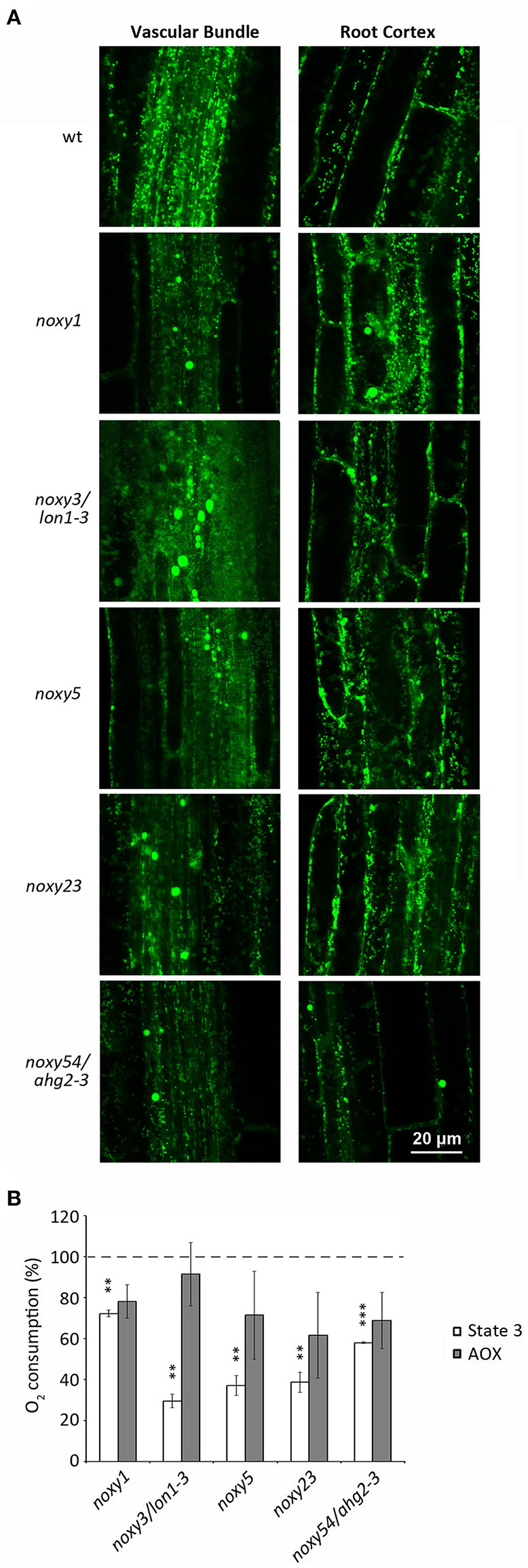
Analysis of mitochondrial distribution and function in wild type, *noxy1, noxy3/lon1-3, noxy5, noxy23*, and *noxy54/ahg2-3* plants. **(A)** Confocal images of mitochondria-tagged mt-YFP in the root vascular bundle (left) and cortex (right) showing abnormal accumulates in *noxy* plants. **(B)** Oxygen consumption rates of cytochrome (State 3) and alternative (AOX) pathways in mitochondria isolated from *noxy* mutants, relative to wild type mitochondria. Asterisks indicate significant differences with wild type plants in each condition (*n* = 4, Student's *t*-test, ^***^P < 0.001, ^**^P < 0.01.

We previously described that both 9-HOT treatment and *noxy* mutations produced abnormal mitochondrial aggregation (Vellosillo et al., 2013). To analyze the mitochondrial morphology of *noxy1, noxy3/lon1-3, noxy5, noxy23*, and *noxy54/ahg2-3*, these mutations were crossed into a transgenic Col-0 line expressing a mitochondria-targeted yellow fluorescent protein (mt-YFP) allowing mitochondrial visualization (Nelson et al., 2007; Vellosillo et al., 2013). Whereas small, individual mitochondria were visible in transgenic Col-0 plants, fluorescence imaging showed abnormal mitochondria accumulates in the roots of all *noxy* mutants. This pattern was more evident in the vascular bundle, where mitochondrial aggregates were accompanied by a noticeable reduction of mitochondrial density in the remaining cells (Figure 2A).

We then addressed whether this abnormal distribution was associated with mitochondrial dysfunction. To this end, we measured the respiratory capacities of mitochondria isolated from *in vitro* grown wild type and *noxy* plants. A significantly reduced cytochrome respiration was detected in all mutants, ranging from ~30% of wild type in *noxy3/lon1-3* to ~70% in *noxy1* (Figure 2B). By contrast, no significant differences were detected in alternative respiration rates (Figure 2B).

### *noxy* Mutants Are Resistant to Antimycin A

**Figure 3:**
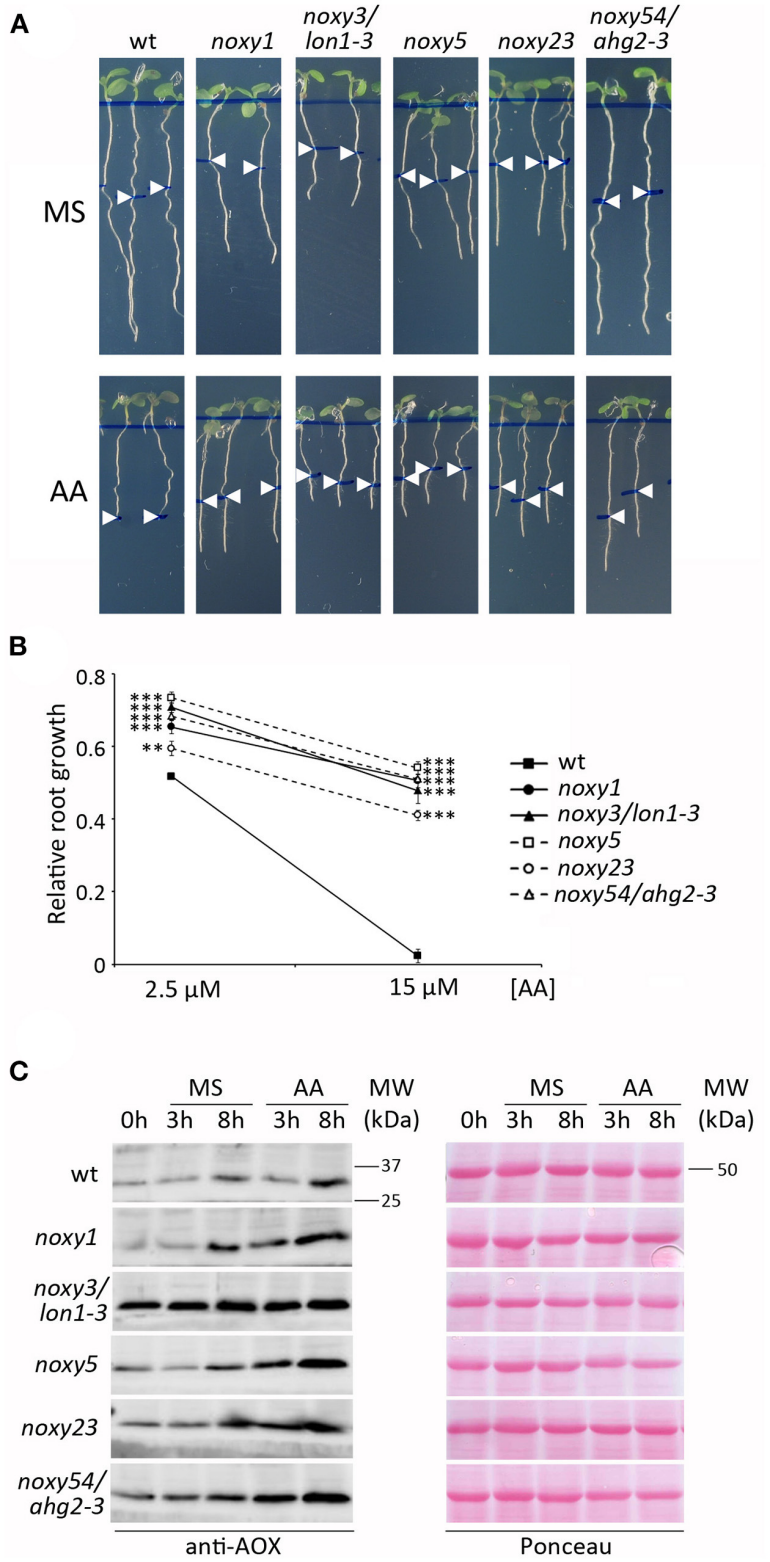
Response of wild type, *noxy1, noxy3/lon1-3, noxy5, noxy23*, and *noxy54/ahg2-3* plants to Antimycin A (AA). **(A)** Root phenotypes of seedlings grown for 3 days on MS medium and then transferred to MS with or without 15 μM AA. Arrow heads indicate root length at the moment of transfer. **(B)** Relative root growth of seedlings in 2.5 and 15 μM AA, defined as the fraction of growth on MS. Asterisks indicate significant differences with wild type plants in each condition (*n* ≥ 8, Student's *t*-test, ^***^P < 0.001, ^**^P < 0.01. **(C)** Western blot showing AOX accumulation in seedlings treated with 20 μM AA. Proteins were extracted and examined at the indicated times. Left: immunoblots with anti-AOX antibody; right: Red Ponceau staining used as loading control. MW, Molecular weight.

Given that all five *noxy* mutations affected mitochondrial functionality, we tested the response of *noxy* mutants to Antimycin A (AA), a widely used and well characterized inhibitor of mitochondrial respiratory complex III (Li et al., 2014). Wild type plants were first exposed to different concentrations of AA to identify a root phenotype allowing subsequent analysis. Root growth reduction was observed with increasing concentrations of AA, until complete growth arrest at 15 μM. Based on this result, AA treatments at 2.5 and 15 μM were selected as inducers of moderate and severe mitochondrial stress, respectively. Growth in AA-containing medium showed that all *noxy* mutants were resistant to both levels of AA; this difference was particularly evident at 15 μM, where complete root growth arrest in wild type plants contrasted with only ~50% reduction in *noxy* mutants (Figures 3A,B). We extended this analysis to previously identified *noxy* mutants with mitochondrial dysfunction (*noxy2, noxy15/drp3a-1, noxy38/fmt-1*, Vellosillo et al., 2013) and the ethylene overproducer *noxy22* (López et al., 2011); in all cases, root growth was not arrested by 15 μM AA (Supplementary Figure 3).

Both AA resistance and 9-HOT insensitivity phenotypes were useful to validate the identity of *noxy* mutations (Supplementary Figure 4). *lon1-4* and *ahg2-1* plants responded similarly to their original mutant alleles *noxy3/lon1-3* and *noxy54/ahg2-3* (Supplementary Figure 4), whereas *noxy23-2* showed an intermediate phenotype compared to *noxy23*, probably due to a lower penetration of the *noxy23-2* mutation (a T-DNA insertion at the promoter region of At1g64880) (Supplementary Figure 4). As no mutant alleles were available for *noxy1* and *noxy5*, we tested the complementation of mutant phenotypes with wild type versions of *NOXY1* and *NOXY5* genes under the 35S promoter. *noxy1*;*35S:NOXY1* plants recovered wild type phenotypes in response to 9-HOT and AA, confirming the identity of *noxy1* mutation. In the case of *noxy5*, phenotype complementation was observed by transformation with wild type *NOXY5* cDNA (Supplementary Figure 4).

In order to overcome AA damage, plants induce alternative oxidases (AOX) to restore electron transport after C-III inhibition (Saisho et al., 1997; Woodson and Chory, 2008). We therefore performed western blot analyses to test whether AOX accumulation in response to AA was affected in *noxy* mutants. In accordance to their resistance to AA, AOX induction was stronger in all *noxy* mutants than in wild type plants. In untreated or control *noxy* plants, AOX levels were also higher than in the wild type. This was particularly evident in *noxy3/lon1-3* plants, which showed constitutively induced AOX expression (Figure 3C).

Altogether, our results indicate that *noxy1, noxy3/lon1-3, noxy5, noxy23*, and *noxy54/ahg2-3* are mutants affected in nucleus-encoded mitochondrial proteins, exhibiting both 9-HOT insensitivity and AA resistance.

### Structurally Related Oxylipins From Different Pathways Modulate Mitochondrial Stress Associated With Complex III

**Figure 4:**
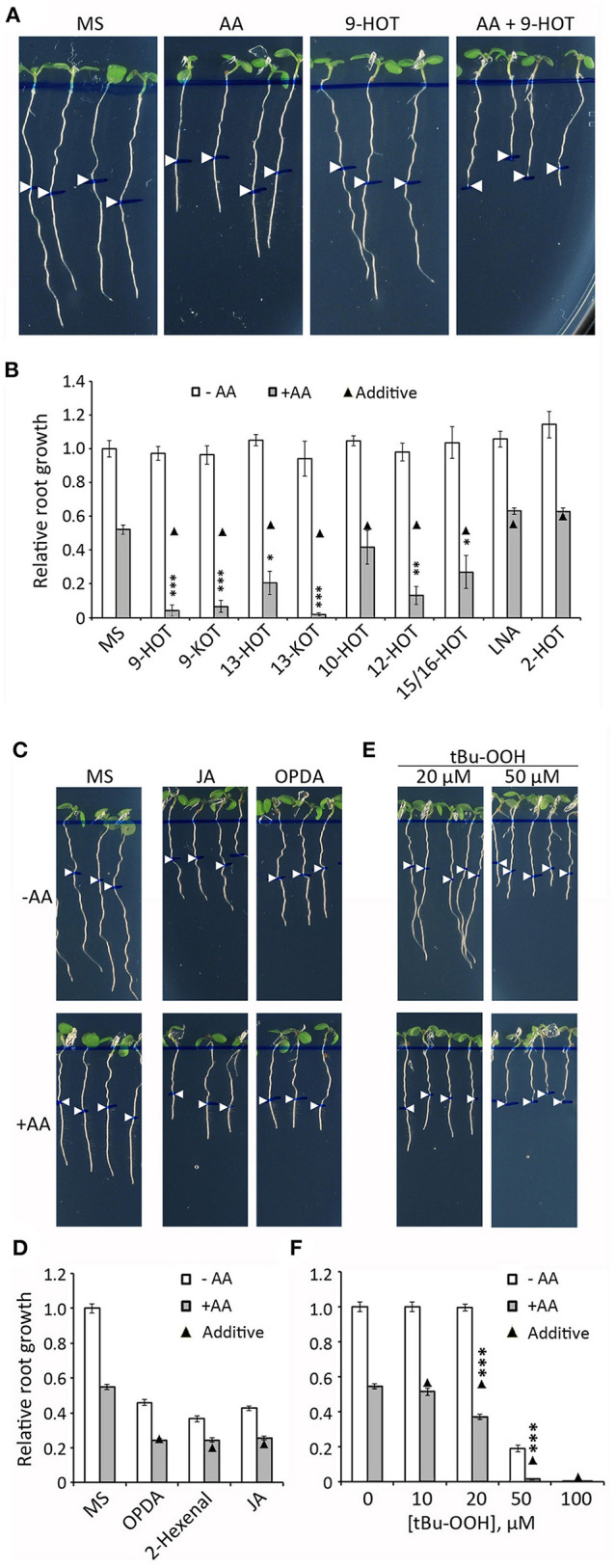
Root growth phenotype in seedlings treated with oxylipins and AA. **(A)** Root phenotypes of wild type plants grown for 4 days on MS medium and then transferred to MS or MS with 2.5 μM AA, 12.5 μM 9-HOT or AA+9-HOT. **(B)** Root growth (relative to MS) of wild type plants in MS, 9-HOT, 9-KOT, 13-HOT, 13-KOT, 10-HOT, 12-HOT, 15/16-HOT, LNA and 2-HOT, alone or in combination with 2.5 μM AA. Oxylipins and LNA were used at 12.5 μM. **(C)** Root phenotypes of wild type plants grown for 3 days on MS medium and then transferred to MS, or to MS with 1 μM OPDA or 10 μM JA, alone or in combination with 2.5 μM AA. **(D)** Root growth (relative to MS) of wild type plants in MS, 1 μM OPDA, 12.5 μM 2-hexenal or 10 μM JA, alone or in combination with 2.5 μM AA. **(E)** Root phenotypes and **(F)** root growth (relative to MS) of wild type plants in different concentrations of tBuOOH, alone or in combination with 2.5 μM AA. In all cases, pictures and measurements were taken on plants 3 days after passing to the indicated media. In **(A,C,E)** root lengths at the moment of medium transfer are pointed by white arrowheads. In **(B,D,F)** black triangles represent expected root growths in combined treatments if both effects were additive (no interaction). Asterisks represent a significant interaction between treatments (*n* ≥ 8, Two way ANOVA, ^***^P < 0.001, ^**^P < 0.01, ^*^P < 0.05).

The coincidence between insensitivity to 9-HOT and AA resistance in *noxy* mutants raised the possibility that both treatments could target a common process, thus we applied both treatments simultaneously and evaluated root phenotypes. A combination of moderate concentrations of 9-HOT (12.5 μM) and AA (2.5 μM) caused complete root growth arrest in wild type plants when added to culture medium (Figure 4A). At these concentrations, 9-HOT alone did not reduce root growth, whereas individual application of AA caused ~50% reduction compared to control seedlings, indicating that 9-HOT amplifies AA-driven root growth reduction. A statistical analysis (two-way ANOVA) confirmed the synergistic interaction between 9-HOT and AA treatments, proving that the root growth reduction observed in the AA/9-HOT combination is stronger to what would be expected from the additive effects of each product (Figure 4B).

We extended this experiment to a collection of oxylipins generated by 9-LOX, 13-LOX, α-DOX and non-enzymatic oxidative pathways, all derived from linolenic acid (LNA) (Figure 1; Supplementary Table 1). Our aim was to identify additional oxylipins that could amplify the response to mitochondrial stress caused by AA (hereafter referred to as mitochondria-active oxylipins). Similar to 9-HOT, amplification of root shortening was observed in AA combined with 9-KOT (from the 9-LOX pathway) as well as with 13-HOT and 13-KOT (13-LOX pathway) (Figure 4B). Plant response to hydroxy fatty acids generated by non-enzymatic pathways varied for different oxylipins. Thus, AA in combination with 12-HOT produced a strong root growth arrest, whereas an intermediate effect was observed with a mixture of 15-HOT/16-HOT, and a marginal response was observed in the combination of AA and 10-HOT. By contrast, LNA and α-DOX-derived 2-HOT did not cause any extra root growth reduction combined with AA (Figure 4B). Amplification of AA root growth arrest was also observed when oxylipin concentration was reduced to 10 μM (with the exception of 10-HOT), however, further reduction to 5 μM caused only a marginal effect (Supplementary Figure 5).

Since 9-KOT and 13-KOT are RES structures, we tested whether other oxylipins bearing α,β-unsaturated carbonyl could amplify the AA-induced root phenotype. The oxylipins 2-hexenal (short chain, linear RES), OPDA (long chain, cyclic RES) and jasmonic acid (non-RES oxylipin control) reduced root growth when applied alone, but their combinations with AA led to an additive response rather than amplification of root growth arrest (Figures 4C,D).

We then examined whether *in vivo* generation of oxylipins by application of tert-butyl hydroperoxide (tBuOOH), an inducer of lipid peroxidation, could amplify AA root growth inhibition. tBuOOH did not arrest root growth when applied at 10 or 20 μM, but reduced root length by ~80% at 50 μM. The combination of tBuOOH with 2.5 μM AA caused a stronger root growth reduction than expected by an additive effect (>60% at 20 μM and complete growth arrest at 50 μM tBuOOH) (Figures 4E,F).

**Figure 5:**
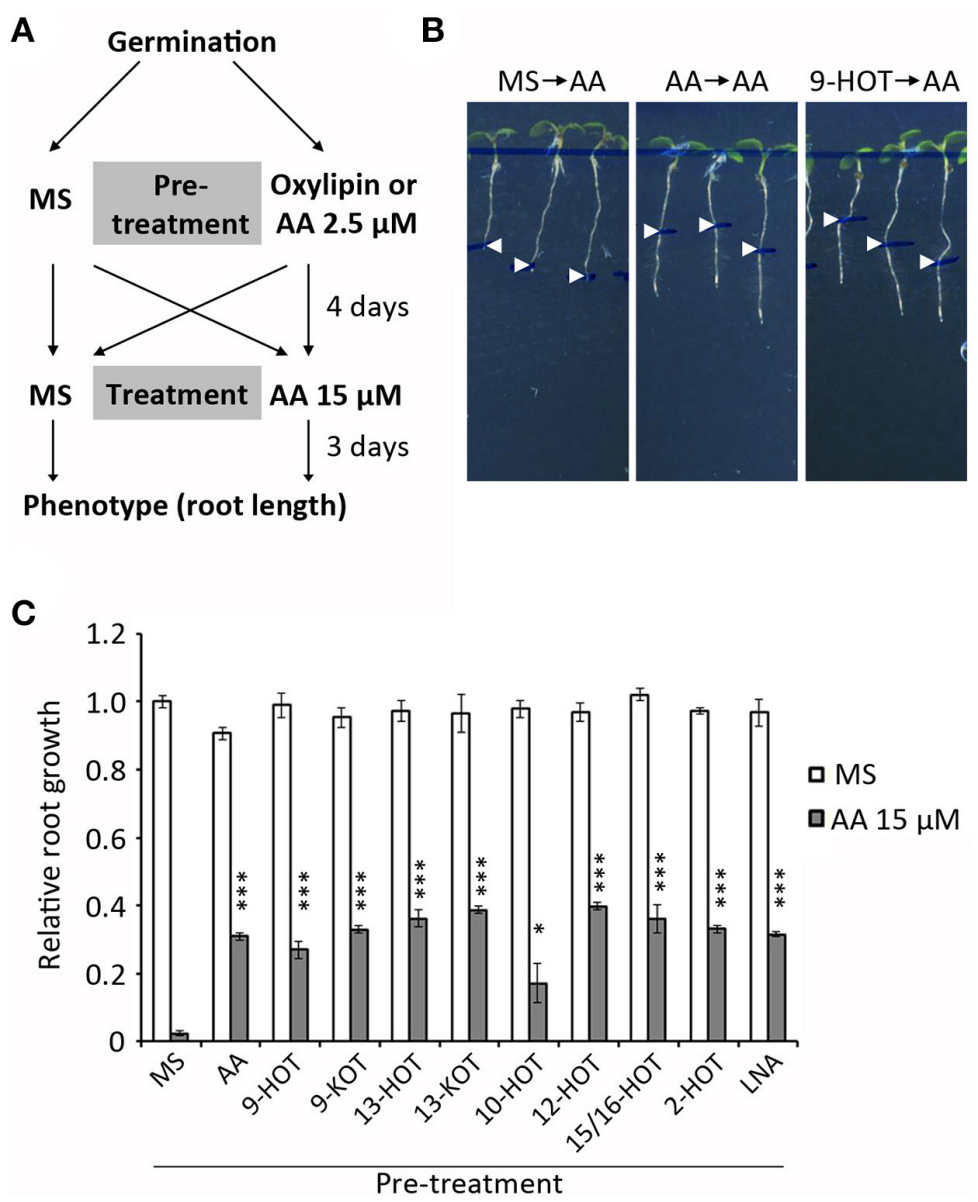
Pretreatment with oxylipins or mild mitochondrial stress protects seedlings against severe mitochondrial damage **(A)** Experiment design: wild type plants were grown 4 days on MS medium with 2.5 μM AA, 20 μM oxylipins (9-HOT, 9-KOT, 13-HOT, 13-KOT, 10-HOT, 12-HOT, 15/16-HOT and 2-HOT) linolenic acid or MS, and then transferred to MS or 15 μM AA to grow for 3 additional days. **(B)** Representative phenotype of plants grown on MS, MS with 2.5 μM AA or 20 μM 9-HOT and transferred to MS with 15 μM AA. Root lengths at the moment of transfer are indicated by arrowheads **(C)** Root growth (relative to plants transferred from MS to MS) of wild type plants transferred from different pre-treatments to MS or MS with 15 μM AA. Asterisks represent significant differences with control plants pre-treated with MS (*n* ≥ 8, Student's *t*-test, ^***^P < 0.001, ^*^P < 0.05).

In order to know if a pre-existing oxylipin signal influences the AA response, we pre-treated wild type plants with oxylipins before AA exposure. Pre-treatments were 25 μM oxylipins, 2.5 μM AA (mild mitochondrial stress control), 25 μM LNA (fatty acid control) and MS (negative control). After 4 days, seedlings were transferred to 15 μM AA or MS and root growth was measured (Figure 5A). As expected, roots of MS pre-treated plants were completely arrested by 15 μM AA. A higher root growth was observed in plants pre-treated with mitochondria-active oxylipins or 2.5 μM AA (~30–40% of MS control, Figure 5). This protection was also observed in plants pre-treated with 10-HOT, 2-HOT, and LNA, suggesting these can be metabolized to mitochondria-active oxylipins.

### Oxylipin Action Is Specific to Complex III, Not Inhibitory and ROS-dependent

**Figure 6:**
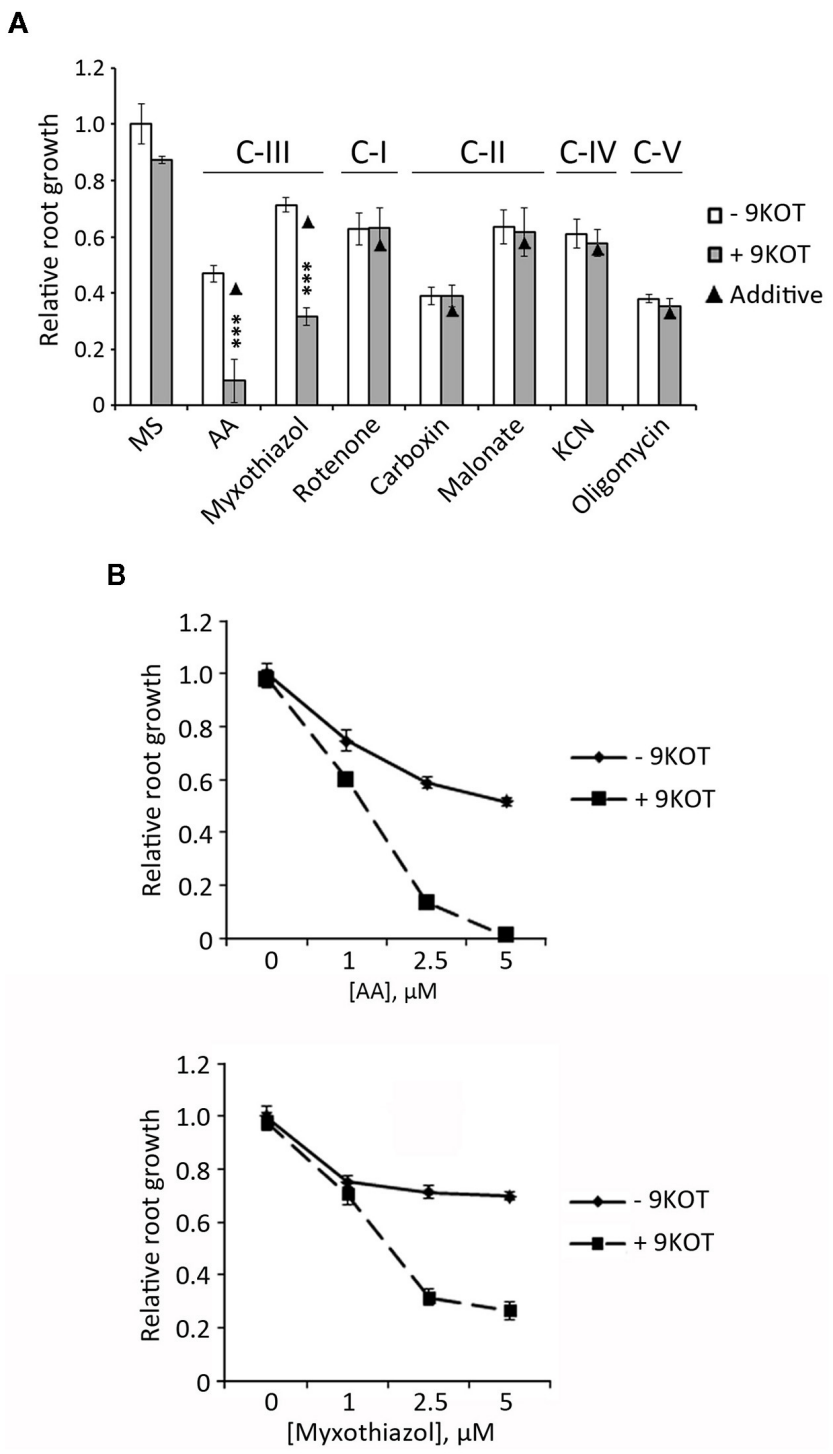
Root growth phenotype in seedlings treated with 9-KOT and inhibitors of respiratory complexes. **(A)** Root growth (relative to MS) of wild type plants grown for 4 days in MS, and then transferred to MS with 2.5 μM AA (C-III), 2.5 μM Myxothiazol (C-III), 50 μM Rotenone (C-I), 100 μM Carboxin (C-II), 2 mM Malonate (C-II), 100 μM KCN (C-IV) or 1 μM Oligomycin A (C-V), alone or in combination with 12.5 μM 9-KOT. Black triangles represent expected root growths in combined treatments if both effects were additive (no interaction). Asterisks represent a significant interaction between treatments (*n* ≥ 8, Two way ANOVA, ^***^P < 0.001. **(B)** Effect on root growth of 12.5 μM 9-KOT in combination with different concentrations of AA and Myxothiazol. Treatments were applied as in **(A)**.

To examine whether oxylipins could also enhance the response to inhibitors of other respiratory complexes, we measured the root length of seedlings grown in 9-KOT combined with Rotenone (complex I), Carboxin (Ubiquinone reductase of complex II), Malonate (Succinate dehydrogenase of complex II), KCN (complex IV), Oligomycin A (complex V), and Myxothiazol (another C-III inhibitor). For comparison with AA control, we selected inhibitor concentrations causing intermediate growth reductions (between 40 and 70%) compared to MS. Figure 6A shows that addition of 9-KOT did not change the root growth reduction caused by inhibitors of complexes I, II, IV or V. In the case of Myxothiazol (C-III inhibitor) root growth reduction was amplified by 9-KOT, although full growth arrest as in the 9-KOT/AA combination was not reached (Figures 6A,B). Hence, oxylipins amplify the respiration inhibition phenotype in a C-III specific manner.

**Figure 7:**
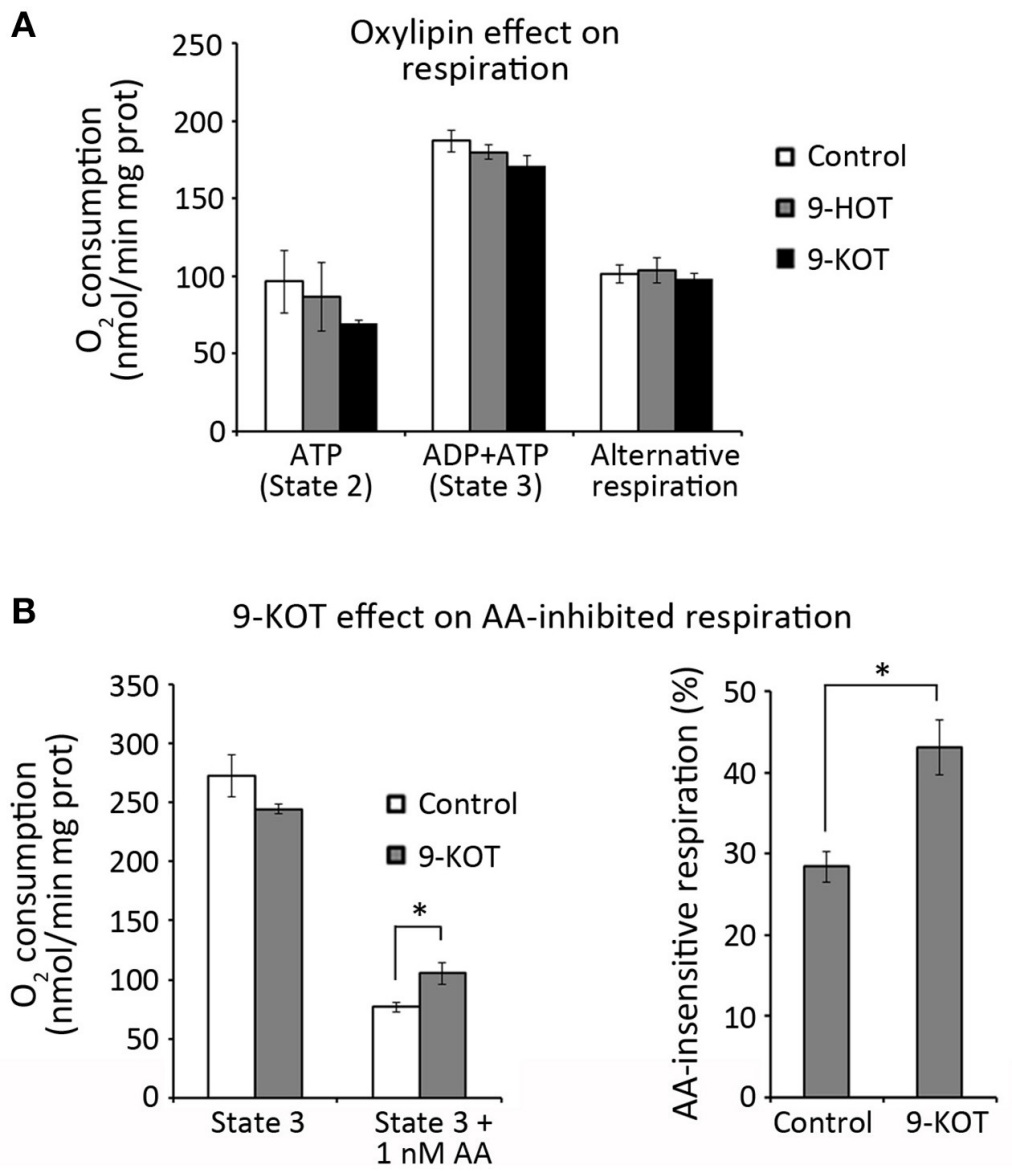
Oxylipin effect on respiration rates of isolated mitochondria. **(A)** O_2_ consumption by the cytochrome (State 2 and State 3) and alternative pathways in the presence of 9-HOT or 9-KOT. **(B)** Effect of 12.5 μM 9-KOT on respiration partially inhibited by 1 nM AA. Left: O_2_ consumption by State 3 mitochondria with or without 9-KOT and AA. Right: Effect of 9-KOT on AA-insensitive respiration, defined as the percent of O_2_ consumed by mitochondria after AA treatment, with or without 9-KOT. Asterisks represent significant differences with controls, as indicated (*n* = 4, Student's *t*-test, ^*^P < 0.05).

These data could be consistent with the action of oxylipins as inhibitors of the cytochrome or alternative respiration pathways. To test this hypothesis, we evaluated the effect of 9-HOT and 9-KOT on the respiratory capacity of isolated mitochondria. Oxygen consumption rates were not changed by these oxylipins in State 2 (with ATP and respiration substrates, but not ADP) or State 3 respiration (after ADP addition), implying that cytochrome respiration was not affected (Figure 7A). Similarly, oxylipin treatment did not affect oxygen consumption after inhibiting the cytochrome pathway while triggering AOX activity, showing that alternative respiration was not affected. We also considered the possibility that oxylipins could inhibit respiration conditionally, that is, only when a C-III inhibitor was present. In our conditions, addition of 1 nM AA inhibited ~70% of oxygen consumption in State 3 (Figure 7B). This value was consistently lower (close to 60%) in the presence of 9-KOT, indicating that instead of reinforcing it, 9-KOT seems to ameliorate AA inhibition of cytochrome respiration capacity. Taken together, these results indicate that 9-HOT and 9-KOT do not seem to act as direct inhibitors of mitochondrial respiration.

**Figure 8:**
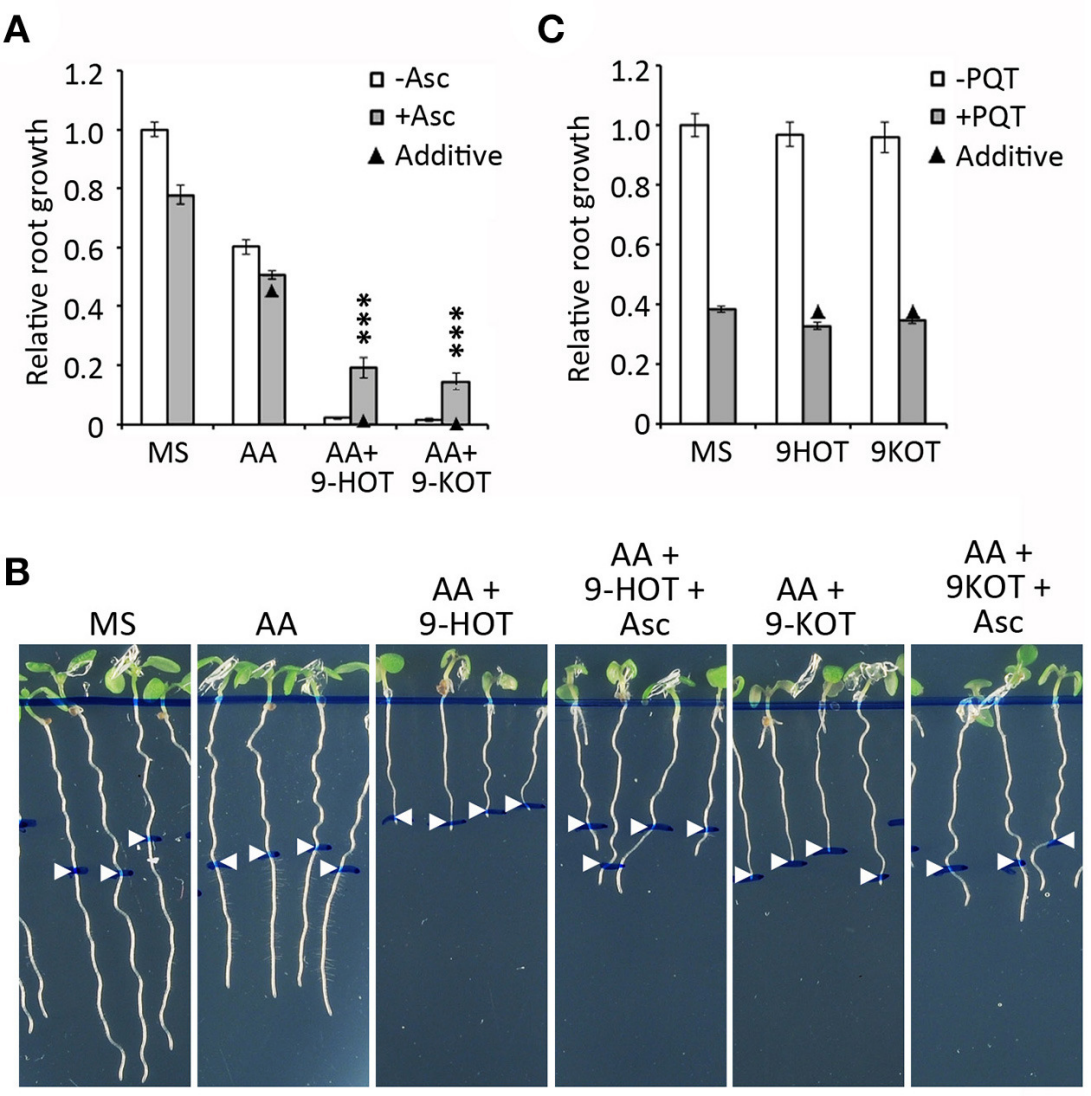
Effect of superoxide on the root growth arrest caused by AA or AA/oxylipin combinations. **(A)** Relative root growth of wild type plants grown for 4 days on MS and then transferred to MS with AA, AA/9-HOT, and AA/9-KOT, with or without the superoxide scavenger Ascorbate (500 μM). **(B)** Representative images of plants from **(A)**. **(C)** Relative root growth of wild type plants in MS, 9-HOT or 9-KOT, with or without addition of the superoxide generator Paraquat (2 nM). In **(A,B)**, AA concentration was 2.5 μM. In all cases, 9-HOT and 9-KOT concentration was 12.5 μM. Black triangles represent expected root growths in combined treatments if both effects were additive (no interaction). Asterisks represent a significant interaction between treatments (*n* ≥ 8, Two way ANOVA, ^***^P < 0.001.

One side effect of AA inhibition of C-III is superoxide generation due to over-reduction of the ubiquinone pool and electron transfer to molecular oxygen (Bleier and Dröse, 2013). In order to know if oxylipins affect ROS production/signaling by C-III, we analyzed the effect of ascorbate (a ROS scavenger) on the root growth arrest phenotype caused by AA/oxylipin mixtures. We first selected 500 μM as the highest concentration of ascorbate at which root growth was not severely affected (data not shown). At this amount, ascorbate did not influence the root growth arrest caused by 2.5 μM AA (Figure 8A). By contrast, it caused a partial reversion of the root growth inhibition produced by AA/9-HOT or AA/9-KOT combinations, indicating that oxylipin action on mitochondria is associated with ROS production (Figures 8A,B). Moreover, 500 μM ascorbate relieved the root arrest caused by high concentrations of AA (10 and 15 μM), but not of KCN, which inhibits respiration without producing superoxide (Supplementary Figure 6). To test whether 9-HOT or 9-KOT enhance the root growth reduction caused by a superoxide source not related to C-III inhibition, we combined these oxylipins with paraquat in the culture media. In our conditions, 2 nM paraquat reduced the root growth of wild type seedlings by ~60%. This value was not changed significantly by addition of 12.5 μM 9-HOT or 9-KOT (Figure 8C), indicating that oxylipin association with ROS production is specific to C-III inhibition.

### Mitochondria-Active Oxylipins Influence Complex III Retrograde Signaling

After mitochondrial damage, nuclear gene expression is activated by retrograde signaling to restore mitochondrial homeostasis. In order to know if oxylipin amplification of AA stress was associated with altered signaling, we analyzed the expression of marker genes of the mitochondrial retrograde pathway (*AOX1A*; Saisho et al., 1997), ROS signaling (transcription factors ZAT12 and WRKY33; Willems et al., 2016; Xu et al., 2017) and 9HOT/9KOT response (ABCG40; López et al., 2011; Marcos et al., 2015). Transcript levels were measured in wild type plants treated for 3 and 8 h with AA, 9-HOT, 9-KOT or AA/oxylipin combinations.

**Figure 9:**
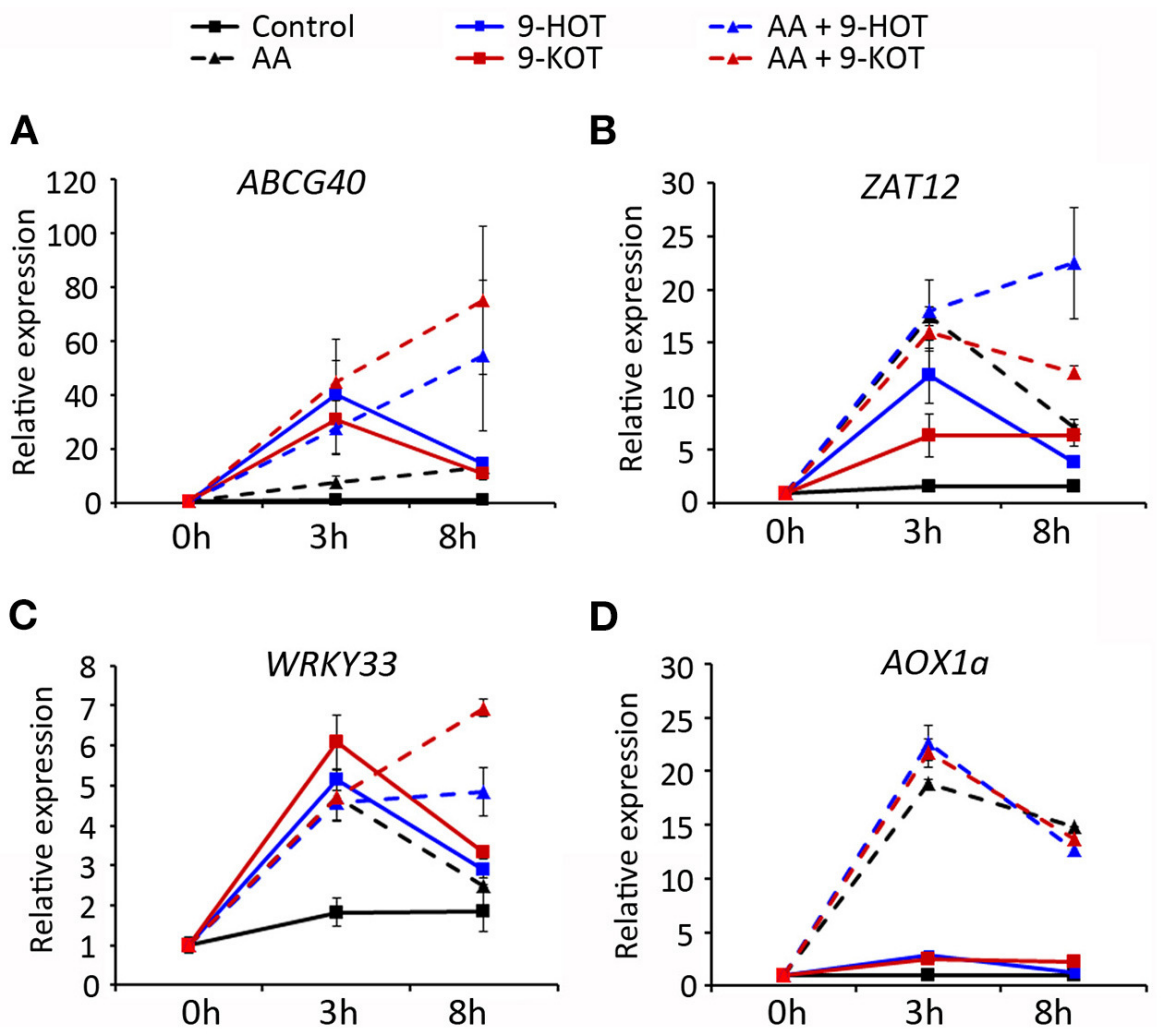
Gene expression in response to 20 μM oxylipins (9-HOT or 9-KOT), 20 μM AA or AA/oxylipin combinations. RNA was extracted and examined at the indicated times after treatment. Selected genes were the oxylipin signaling marker *ABCG40* **(A)**, ROS-specific transcription factors *ZAT12* **(B)** and *WRKY33* **(C)** and the mitochondria retrograde signaling marker *AOX1A* **(D)**. Expression values are relative to untreated plants, using 18S rRNA and *SAND* as reference genes.

*ABCG40* was strongly induced (~30–40-fold) 3 h after treatment with oxylipins alone or in combination with AA, whereas the application of AA alone caused a milder induction (8-fold). At 8 h, expression increased slightly in AA-treated plants (13-fold) but decreased in oxylipin treatments (to ~10–15-fold). By contrast, AA/oxylipin combinations amplified *ABCG40* expression 8 h after treatment, with transcript levels reaching 50 and 70-fold in response to AA/9-HOT and AA/9-KOT, respectively (Figure 9A). A similar pattern was observed for *WRKY33* and *ZAT12* transcripts, which expression increased at 3 h after treatment with AA, 9-HOT or 9-KOT, and later descended in 8 h samples. In response to AA/9-HOT or AA/9-KOT combinations, induction of both genes was sustained, and transcript levels at 8 h were higher than in single AA or oxylipin treatments (Figures 9B,C). As expected, the level of *AOX1A* transcripts increased in response to AA treatment (20 and 15-fold at 3 and 8 h, respectively). Application of 9-HOT or 9-KOT, either alone or in combination with AA, did not markedly change *AOX1A* expression (Figure 9D).

### *noxy* Mutants Are More Tolerant to Oxidative Stress

**Figure 10:**
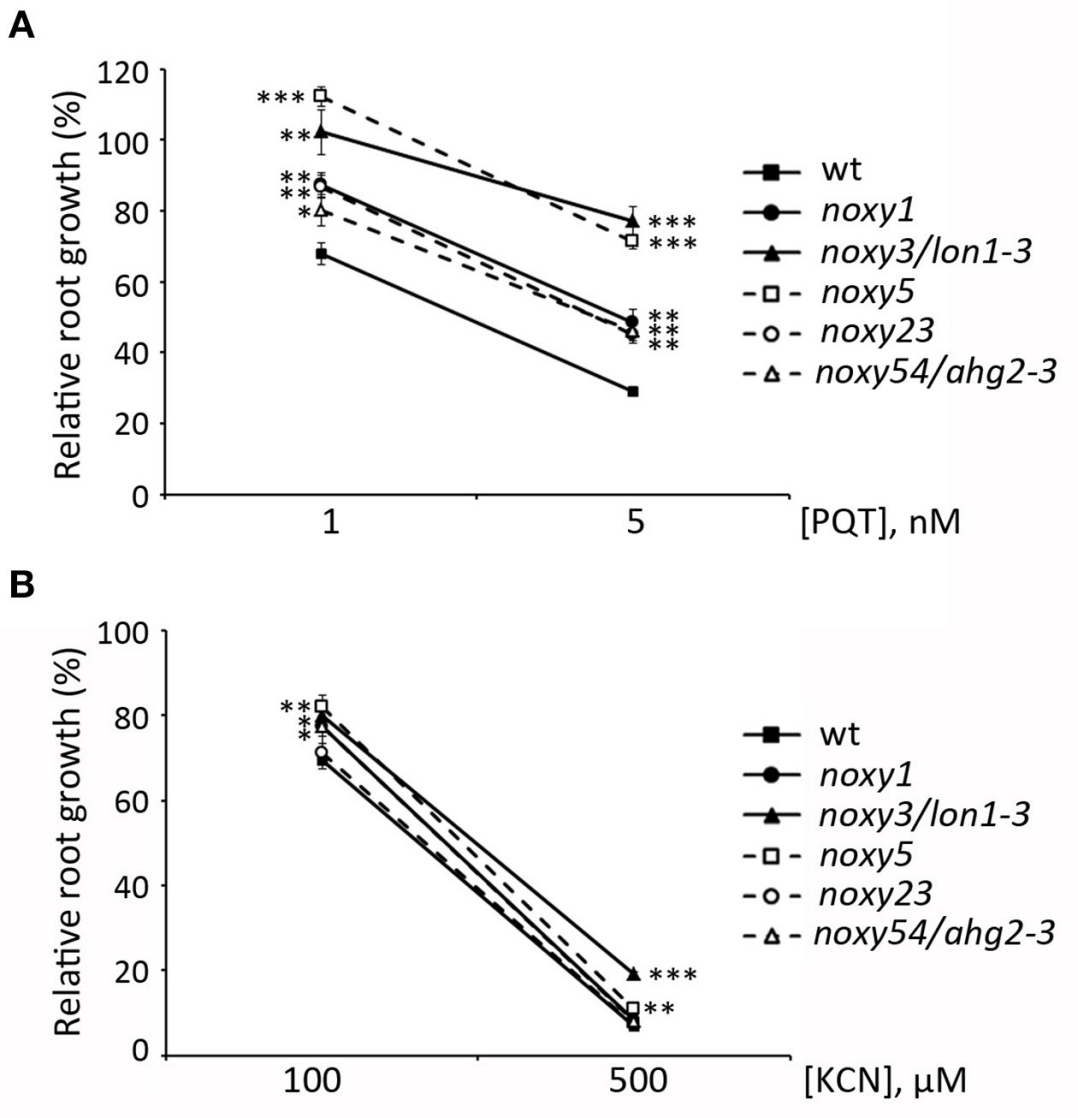
Effect of ROS-generating Paraquat (PQT) and non-ROS-generating KCN on the root growth of *noxy* mutants. Relative root growth of wild type and *noxy1, noxy3/lon1-3, noxy5, noxy23*, and *noxy54/ahg2-3* plants in Paraquat **(A)** and KCN **(B)**. Asterisks indicate significant differences with wild type plants in each condition (*n* ≥ 8, Student's *t*-test, ^***^P < 0.001, ^**^P < 0.01, ^*^P < 0.05).

Our results indicate that oxylipin action on mitochondria could be primarily associated with superoxide production. Accordingly, insensitivity of *noxy* plants to oxylipins could be accompanied by enhanced resistance to this type of ROS. To test this possibility, we examined the root phenotypes of *noxy* plants grown in paraquat-containing medium. In wild type plants, addition of this herbicide at concentrations 1 and 5 nM reduced root growth by ~30 and ~70%, respectively. Compared to these values, root growth reduction was milder in all *noxy* mutants, which thus appear partially resistant to superoxide damage (Figure 10A). Conversely, KCN is an inhibitor of cytochrome respiration that does not induce ROS production (Chen et al., 2003). In contrast to AA, *noxy* mutants responded like wild type plants to KCN at the two concentrations tested (100 and 500 μM), showing only minor differences probably due to specific compensation mechanisms (Figure 10B).

## Discussion

Oxylipins are a diverse group of oxidized lipid derivatives with relevant signaling activities in developmental and stress responses. However, precise assignment of their functions is challenging due to multiple enzymatic and non-enzymatic sources, biosynthetic genes and metabolic interaction of the existing pathways (summarized in Figure 1). Here, we report that oxylipins generated from 9-LOX, 13-LOX and non-enzymatic pathways converge as enhancers of the stress response produced by Antimycin A, a ROS-generating inhibitor of mitochondrial complex III.

### *noxy* Mutants and Oxylipin Function

The identification of five *noxy* mutations (*noxy1, noxy3, noxy5, noxy23*, and *noxy54*) in nuclear genes encoding mitochondrial proteins supports the participation of mitochondria in 9-LOX-derived oxylipin signaling as described (Vellosillo et al., 2013). As the majority of mitochondrial proteins are nuclear-encoded, any perturbation in this organelle needs to be signaled to the nucleus (retrograde signaling) to modulate gene expression and restore mitochondrial homeostasis (Rhoads and Subbaiah, 2007). The inhibition of C-III by AA is a mitochondrial stress model that has allowed the identification of key components of the retrograde pathway such as the transcription factor ANAC17 and the protein kinase CDKE1, both mediating the induction of AOX proteins to restore electron transport (Ng et al., 2013a,b). The *noxy* mutations described here alter mitochondrial morphology and respiration rates, which is consistent with higher AOX levels in these *noxy* mutants before and/or after AA treatment. These alterations are indicative of mitochondrial stress likely caused by altered mitochondrial RNA or protein homeostasis, as suggested by the functions of the mutated genes. Accordingly, *ahg2-1* affects C-III levels (Hirayama et al., 2013) and *lon1-1* impairs the accumulation of electron transport chain (ETC) components (Solheim et al., 2012). Likewise, *noxy1* and *noxy5* mutations in PPR proteins could lead to aberrant RNA editing, whereas *noxy23* could result in impaired mitochondrial protein synthesis, ultimately leading to mitochondrial stress. This rationale can also be applied to previously described *noxy* mutants with abnormal mitochondria, which are also AA-resistant (Vellosillo et al., 2013 and Supplementary Figure 3). Since 9-HOT and 9-KOT are associated to mitochondrial stress, *noxy* mutant insensibility to these oxylipins could be a consequence of upregulated mitochondrial retrograde signaling. Interestingly, other *noxy* mutations not directly associated with mitochondria, such as *noxy7/gcn1-3, gcn20-2* (general translation control) and *noxy22* (ethylene signaling) also confer resistance to AA (López et al., 2011; Izquierdo et al., 2018 and Supplementary Figure 3) making non-mitochondrial *noxy* mutants an interesting tool to search for new mitochondrial-nucleus communication pathways.

9-HOT induces mitochondria aggregation and decreases inner membrane potential (Vellosillo et al., 2013). Similarly to moderate AA (2.5 μM) pre-treatment, this mild mitochondrial stress protects plants against subsequent, root-arresting AA levels. Oxylipin functions could therefore include the induction of retrograde signals to protect plants against mitochondrial damage. It is intriguing how, alongside this protective role, mitochondria-active oxylipins potentiate AA damage when applied simultaneously. This opens the question of whether natural C-III inhibitors exist in plants, whose action would be enhanced by endogenously produced oxylipins. For instance, salicylic acid has been proposed to inhibit C-III and activate C-II to induce mitochondrial ROS production (Nie et al., 2015; Belt et al., 2017), which could be linked to oxylipin roles in plant defense.

9-HOT and 9-KOT amplification of AA root phenotype was accompanied by induction of stress-responsive genes, illustrating the activation of retrograde signaling pathways. Thus, sustained induction of *ABCG40* (9-HOT responsive gene), *ZAT12* and *WRKY33* (ROS-responsive transcription factors) suggests that oxylipin-triggered mitochondrial signals mediate the stress responses reportedly associated with these genes (Davletova et al., 2005; Zheng et al., 2006; Borghi et al., 2015). Interestingly, induction of *AOX1a* by AA is not affected by these oxylipins. As this gene is considered the hallmark of the retrograde pathway (Woodson and Chory, 2008; Ng et al., 2013b), oxylipin retrograde signaling could be independent of ANAC17 and CDKE1.

### Mitochondria-Active Oxylipins Affect Complex III-Derived ROS Signaling

Evidence points to C-III as the primary target of a vast array of oxylipins derived from 9-LOX, 13-LOX and non-enzymatic pathways, presenting the challenge to understand how this common feature fits into the negative and positive interactions among oxylipin pathways (Vicente et al., 2012; Zoeller et al., 2012; Wang et al., 2020). Instead of being *bona fide* C-III inhibitors, our results indicate that mitochondria-active oxylipins might act by enhancing ROS production and signaling by this complex. A first indication was that 9-KOT arrests root growth in combination with AA but not with KCN, both inhibitors of cytochrome respiration which differ in ROS production. Secondly, root arrest caused by AA/9-HOT and AA/9-KOT combinations can be partially reversed by the superoxide scavenger ascorbate. An increment of superoxide production by AA/oxylipins is also consistent with the higher oxygen consumption rate observed in mitochondria treated with AA/9-KOT, compared to AA alone. Nevertheless, specific measurements or imaging analyses will be needed to determine specific ROS contribution to oxylipin signaling.

How mitochondria-active oxylipins act on C-III remains an open question. Based on their chemical structure, they are α,β-unsaturated keto acids (9-KOT and 13-KOT) or hydroxy acids (9,12,13,15 and 16-HOT). The former are RES that could bind directly to cysteine residues, whereas oxidation of the latter to RES structures is thermodynamically favored (House et al., 1972). It is therefore tempting to speculate that oxylipins could bind covalently to their targets to promote C-III ROS production. In basal conditions, about 1–5% of ETC electrons are “lost” at C-III by collateral superoxide generation (Osyczka et al., 2005). This rate increases after AA inhibition, as well as in response to environmental stress conditions such as hypoxia, salt, drought and pathogen attack (Mittova et al., 2003; Rhoads et al., 2006; Cruz De Carvalho, 2008; Colombatti et al., 2014; Wagner et al., 2018), all situations in which oxylipin production has been reported (Blée, 2002; Ghanem et al., 2012; Moradi et al., 2017; Savchenko et al., 2019). Superoxide can generate a second wave of highly oxidant ROS, causing extensive oxidation of lipids and proteins and ultimately cell death. In this scenario, the oxidative stress caused by high AA, or low AA combined with mitochondria-active oxylipins, could exceed the cell antioxidant capacity leading to full growth arrest. Alternatively, oxylipins could enhance C-III superoxide production indirectly through modulation of AOX activity. Although we did not detect any influence of 9-HOT or 9-KOT on the alternative respiration capacity of isolated mitochondria, we cannot rule out that AOX activity could be affected *in vivo* by mitochondria-active oxylipins.

## Conclusion

Taken together, our results show that oxylipins from different pathways converge as activators of mitochondrial retrograde signaling through complex III, potentially controlling cell responses to a wide range of stress conditions. Future studies will be needed to determine how this pathway influences plant acclimation and fitness.

## Data Availability Statement

The original contributions presented in the study are included in the article/Supplementary Material, further inquiries can be directed to the corresponding authors.

## Author Contributions

CC, YI, and LM designed the study. YI, JV, SK, VA, and TC mapped the *noxy* mutations. LM and AM-A generated transgenic *noxy* lines. YI, LM, and BL performed *in vitro* phenotypic analyses. YI and CC generated confocal images. YI, AL and CC wrote the paper. YI, TC, and AL conducted gene expression analyses. YI performed respiration measurements. All authors read and approved the final version of the manuscript.

## Conflict of Interest

SK conducted his research work at Centro Nacional de Biotecnología and is currently employed by GlaxoSmithKline. The remaining authors declare that the research was conducted in the absence of any commercial or financial relationships that could be construed as a potential conflict of interest.

## Publisher's Note

All claims expressed in this article are solely those of the authors and do not necessarily represent those of their affiliated organizations, or those of the publisher, the editors and the reviewers. Any product that may be evaluated in this article, or claim that may be made by its manufacturer, is not guaranteed or endorsed by the publisher.
