# Supplementary material for: Oxylipins From Different Pathways Trigger Mitochondrial Stress Signaling Through Respiratory Complex III
Source: Front Plant Sci. 2021 Jul 29;12:705373. doi: 10.3389/fpls.2021.705373 (PMC8358658; doi:10.3389/fpls.2021.705373)
Supplement: Supplementary file 1 [file Data_Sheet_1.pdf]

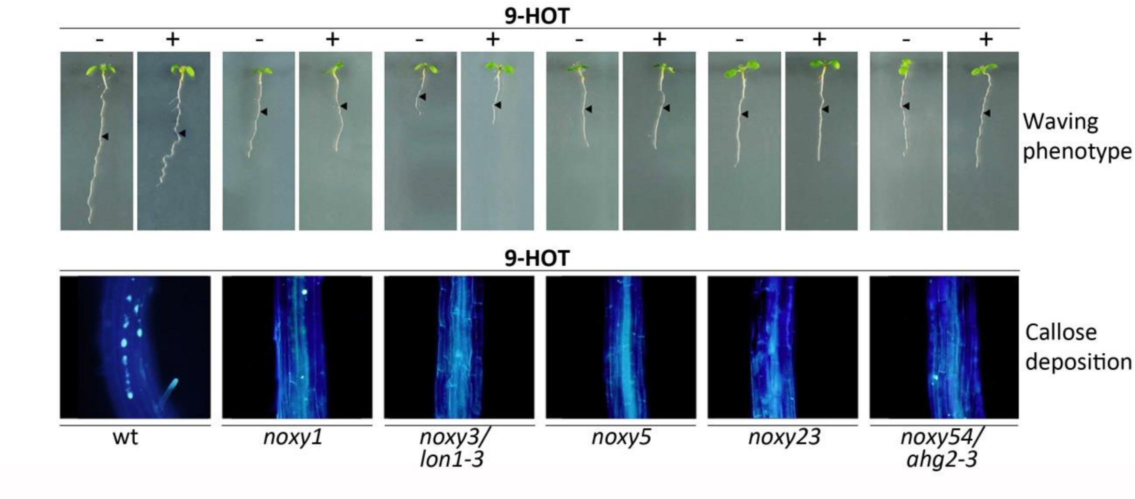

Supplemental Figure 1. 9-HOT responses in wild type and *noxy* plants. Upper panel: root waving phenotype in plants grown for three days on MS medium and then transferred to MS or 25 μM 9-HOT. Length of roots at the moment of transfer is indicated by arrowheads. Lower panel: callose accumulation in roots of 9-HOT-grown plants.

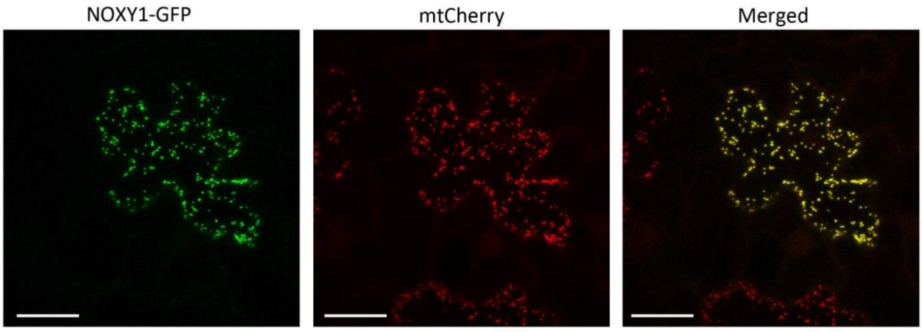

Supplemental Figure 2. Localization of NOXY1:GFP in *N. bentmainana*. Images of NOXY1:GFP fusion protein (green), and mitochondria-specific mtCherry (red) transiently expressed in *N. benthamiana* leaves. Merged image shows colocalization in mitochondria (yellow). Bar = 20 μm.

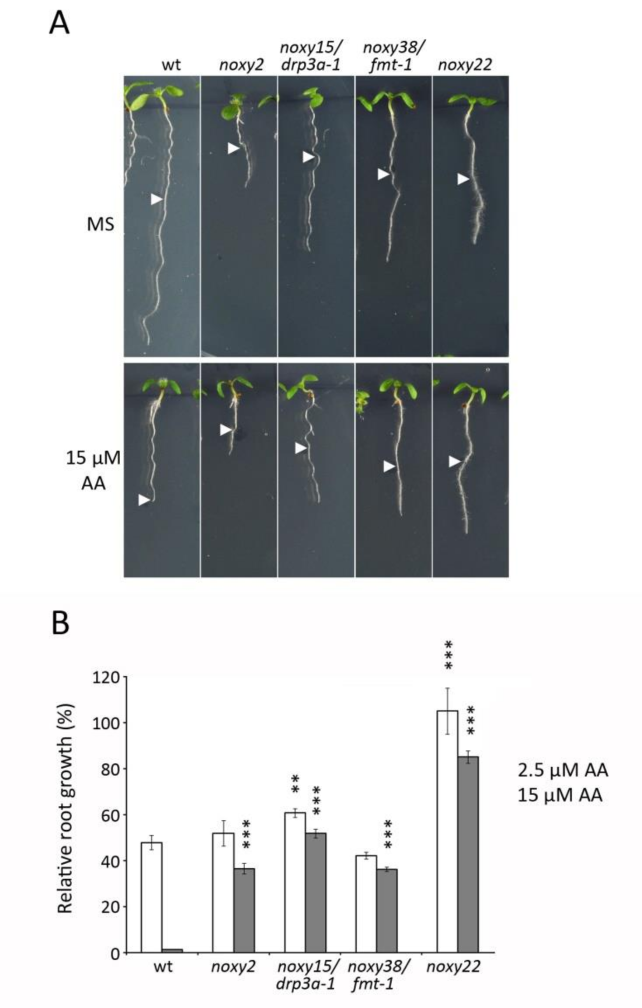

Supplemental Figure 3. Response to AA of mutants *noxy2*, *noxy15/drp3a-1*, *noxy38/fmt-1* and *noxy22*. **(A)** Representative phenotype of wild type and *noxy* plants grown for three days on MS medium and then transferred to MS with or without 15 µM AA. Length of roots at the moment of transfer is indicated by arrowheads. **(B)** Measurement of root lengths (relative to MS) of seedlings after transfer to MS medium with 2.5 µM or 15 µM AA. Asterisks indicate significant differences with wild type plants in each condition (n >= 8, Student's t-test, *** $P < 0.001$, ** $P < 0.01$).

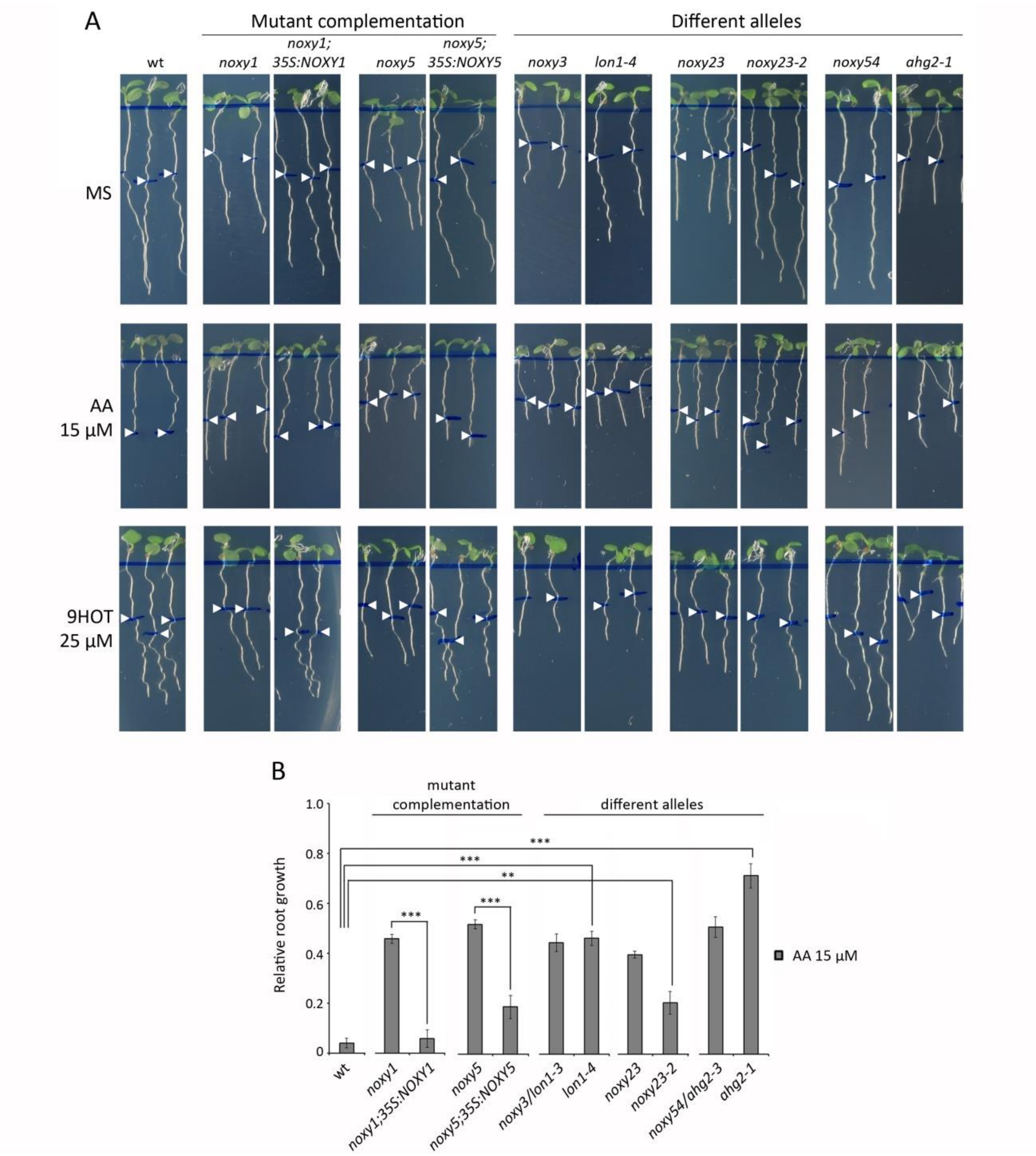

Supplemental Figure 4. Validation of *noxy* mutations identity by phenotypic analysis. **(A)** Phenotypes of wild type plants, *noxy* mutants, *noxy1* and *noxy5* transformed with wild type versions of *NOXY1* and *NOXY5* and mutant alleles *lon1*-2, *noxy23-2* and *ahg2-1* grown on MS (upper panel), 15 µM AA (mid panel) or 25 µM 9-HOT (lower panel). Length of roots at the moment of transfer is indicated by arrowheads. **(B)** Measurement of root lengths (relative to MS) of AA-grown seedlings shown in (A). Asterisks indicate significant differences as indicated. (n >= 8, Student's t-test, *** $P < 0.001$, ** $P < 0.01$). For complementation lines, statistical tests were performed between these and their parental *noxy* mutants; for alternative mutant alleles, this was done between these and wild type plants.

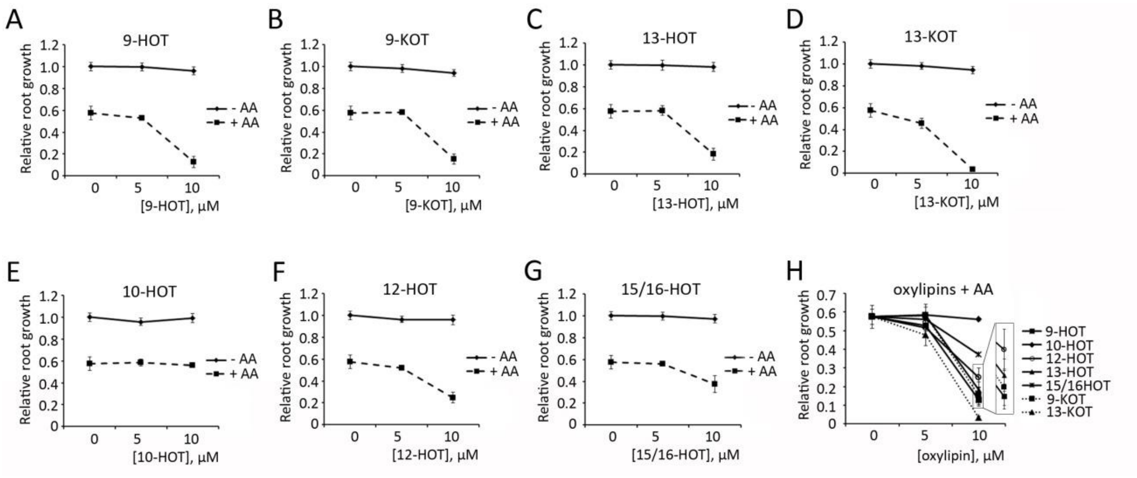

Supplemental Figure 5. Concentration-dependent effect of mitochondria-active oxylipins on the root growth reduction caused by 2.5 μM AA. Root growth (relative to MS) in 9-HOT **(A)**, 9-KOT **(B)**, 13-HOT **(C)**, 13-KOT **(D)**, 10-HOT **(E)**, 12-HOT **(F)** and 15/16-HOT **(G)** alone or in combination with 2.5 μM AA. For comparison, AA/oxylipin combinations are summarized in **(H)**.

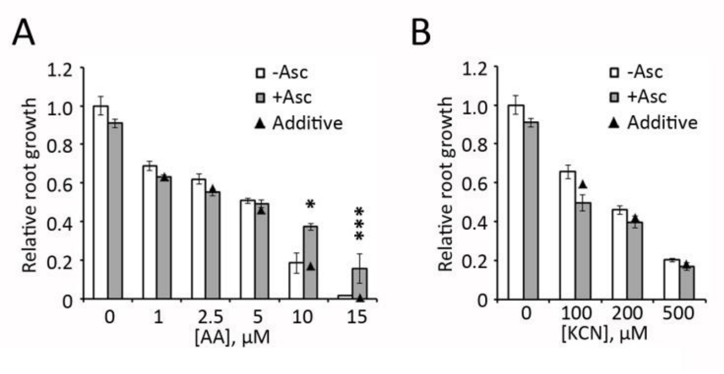

Supplemental Figure 6. Effect of ascorbate on the root growth reduction caused by ETC inhibitors. **(A)** Measurement of root lengths (relative to MS) of wild type plants grown in increasing concentrations of AA, with or without 500 μM Ascorbate. **(B)** Measurement of root lengths (relative to MS) of wild type plants grown in increasing concentrations of KCN, with or without 500 μM Ascorbate. Black triangles represent expected root growths in combined treatments if both effects were additive (no interaction). Asterisks represent a significant interaction between treatments (n >= 8, Two way ANOVA, *** $P < 0.001$, * $P < 0.05$).
