# Supplementary material for: Oxylipins From Different Pathways Trigger Mitochondrial Stress Signaling Through Respiratory Complex III
Source: Front Plant Sci. 2021 Jul 29;12:705373. doi: 10.3389/fpls.2021.705373 (PMC8358658; doi:10.3389/fpls.2021.705373)
Supplement: Supplementary file 2 [file Table_1.DOCX]

| Supplemental Table 1. Oxylipins used in this study | |
| --- | --- |
| Short name | IUPAC name |
| 2-HOT  9-HOT  10-HOT  12-HOT  13-HOT  15/16-HOT  9-KOT  13-KOT  2-Hexenal  OPDA  Jasmonic acid | 2(*R*)-hydroxy-9(*Z*),12(*Z*),15(*Z*)-octadecatrienoic acid  9(*S*)-hydroxy-10(*E*),12(*Z*),15(*Z*)-octadecatrienoic acid  10(*R,S*)-hydroxy-8(*E*),12(*Z*),15(*Z*)-octadecatrienoic acid  12(*R,S*)-hydroxy-9(*Z*),13(*E*),15(*Z*)-octadecatrienoic acid  13(*S*)-hydroxy-9(*Z*),11(*E*),15(*Z*)-octadecatrienoic acid  Mixture of 15(*R,S*)-hydroxy-9(*Z*),12(*Z*),16(*E*)-octadecatrienoic acid and 16(*R,S*)-hydroxy-9(*Z*),12(*Z*),14(*E*)-octadecatrienoic acid (comparable amounts)  9-keto-10(*E*),12(*Z*),15(*Z*)-octadecatrienoic acid  13-keto-9(*Z*),11(*E*),15(*Z*)-octadecatrienoic acid  2(*E*)-hexenal  12-oxo-10,15(*Z*)-phytodienoic acid  2-[(1*R*,2*R*)-3-oxo-2-[(*Z*)-pent-2-enyl]cyclopentyl]acetic acid |
