# Supplementary material for: Oxylipins From Different Pathways Trigger Mitochondrial Stress Signaling Through Respiratory Complex III
Source: Front Plant Sci. 2021 Jul 29;12:705373. doi: 10.3389/fpls.2021.705373 (PMC8358658; doi:10.3389/fpls.2021.705373)
Supplement: Supplementary file 3 [file Table_2.DOCX]

| Supplemental Table 2. qPCR primers used in this study | |  |
| --- | --- | --- |
| Target gene (AGI code) | Forward primer | Reverse primer |
| 18S rRNA  *SAND* (At2g28390)  *ZAT12* (At5g59820)  *WRKY33* (At2g38470)  *ABCG40* (At1g15520)  *AOX1a* (At3g22370) | 5’ GTCTTAATTGGCCGGGTCGTG  5’ CAAGGCAGGAAATCACCAGGTTG  5’ ATGTCACGGCGGCGAATTGT  5’ TGGAGAGAGCATCACACGACA  5’ GGTTTTGCCAGCTCCAGAGA  5’ TTGTTCTTCCAGAGGAGATA | 5’ CGAAGGCCAACACAATAGGATCG  5’ CTGTACAGCTGATGCAGACCAG  5’ CCTCCTAAGGCTTGGAACGA  5’ TCTGTTTGTGGCGTAACCGC  5’ CTCAGTTGACATGCCCCAGGAA  5’ CTCAAAACGTCGAAGCGA |
